# Comparative accuracy of CT perfusion in diagnosing acute ischemic stroke: A systematic review of 27 trials

**DOI:** 10.1371/journal.pone.0176622

**Published:** 2017-05-17

**Authors:** Jiantong Shen, Xianglian Li, Youping Li, Bing Wu

**Affiliations:** 1 Key Laboratory of Transplant Engineering and Immunology of the Ministry of Health of China, West China Hospital, Sichuan University, Chengdu, PR China; 2 Chinese Cochrane Center, West China Hospital, Sichuan University, Chengdu, PR China; 3 Department of Radiology, West China Hospital, Sichuan University, Chengdu, PR China; "INSERM", FRANCE

## Abstract

**Objective:**

To systematically evaluate and compare the diagnostic accuracy of CT perfusion (CTP), non-enhanced computed tomography (NCCT) and computed tomography angiography (CTA) in detecting acute ischemic stroke.

**Methods:**

We searched seven databases and screened the reference lists of the included studies. The risk of bias in the study quality was assessed using QUADASII. We produced paired forest plots in RevMan to show the variation of the sensitivity and specificity estimates together with their 95% CI. We used a hierarchical summary ROC model to summarize the sensitivity and specificity of CTP in detecting ischemic stroke.

**Results:**

We identified 27 studies with a total of 2168 patients. The pooled sensitivity of CTP for acute ischemic stroke was 82% (95% CI 75–88%), and the specificity was 96% (95% CI 89–99%). CTP was more sensitive than NCCT and had a similar accuracy with CTA. There were no statistically significant differences in the sensitivity and specificity between patients who underwent CTP within 6 hours of symptom onset and beyond 6 hours after symptom onset. No adverse events were reported in the included studies.

**Conclusions:**

CTP is more accurate than NCCT and has similar accuracy to CTA in detecting acute ischemic stroke. However, the evidence is not strong. There is potential benefit of using CTP to select stroke patients for treatment, but more high-quality evidence is needed to confirm this result.

## Introduction

Stroke is the second leading cause of death and the third leading cause of disability in the world [1, 2]. In 2010, there were 16.9 million incident stroke cases, 33.0 million prevalent stroke cases, 5.9 million deaths attributed to stroke, and 102.2 million lost DALYs [3,4]. According to a report from the American Heart Association, approximately 87% of all strokes were ischemic strokes [5]. An accurate and timely diagnosis of ischemic stroke is crucial for establishing an appropriate patient treatment. Non-enhanced computed tomography (NCCT) is widely used in acute ischemic stroke imaging due to its rapid performance, high tolerance, and high reliability [6]. However, NCCT has difficulty in detecting early infarct signs and is influenced by the size of the infarct and severity of ischemia. CT perfusion (CTP) is performed for physiological evaluation of the brain parenchyma, which allows better detection of ischemia. However, the accuracy of CTP in detecting acute ischemic stroke (AIS) was still uncertain. To the best of our knowledge, there were two systematic reviews to evaluate the diagnostic value of CTP for AIS. A systematic review which based on 15 studies showed that the sensitivity and specificity of CTP were 80% and 95% respectively; however, another systematic review which based on 11 studies reported that the sensitivity and specificity of CTP were 69.9% and 87.7% [7,8]. These conflicting results revealed the possibility of publication bias and omission of critical studies. Taking into account the limitations of the previously mentioned reviews and the appearance of new evidence, there is still a need to evaluate how well CTP detects acute ischemic stroke. The purpose of the present study is to review the accuracy of CTP in detecting ischemic stroke systematically, and at the same time, to compare accuracy of CTP with either NCCT or computed tomography angiography (CTA) in detecting ischemic stroke systematically.

## Methods

### Criteria for considering studies for this review

The studies were eligible that met the following inclusion criteria. 1) The studies either focused on CTP or compared CTP with other imaging methods. 2) The absolute numbers of the observations of true positives, false positives, false negatives, and true negatives were either available or derivable from the data which was reported in the primary studies. 3) Human subjects were the focus of analysis. 4) The studies were either prospective or retrospective.

We excluded the following studies that addressed specific anatomical, metabolic, or microvascular aspects of stroke; focused on the specific technical parameters of CTP; location of final infarct; distinguished ischemic core from the salvageable brain tissue. If several reports were based on the same study, we selected the most recent or most complete publication available.

### Literature search

We performed an initial literature search of PubMed, EMBASE, CENTRAL, the Cochrane Database of Systematic Reviews, the Cochrane Controlled Trials Register Database of Reviews of Effectiveness, and Health Technology Assessment database in June 2015 and updated search in June 2016. We also scanned the references of all articles which were selected for the review to find any potentially relevant articles. The search strategy was list in S1 Table.

### Study process

Two reviewers (JTS and XLL) who were trained in research methods screened all titles/abstracts and full texts to select articles that met the inclusion criteria independently. The same two reviewers also assessed the risk of bias independently and extracted information for each included study. Reviewers dealt with discrepancies through discussion.

### Assessment of methodological quality

We used the quality assessment of diagnostic accuracy studies II (QUADASII) to assess the methodological quality of each included study [9]. QUADASII was updated in 2010 that consists of four domains as follows: patient selection, index test, reference standard and patient flow. Each domain was assessed in terms of the risk of bias, and the first three domains were also considered in terms of applicability. Clinical assessment with follow up imaging (MRI or CT) was defined as a reference standard to classify a stroke diagnosis correctly.

### Data collection

We extracted information from each included study, and these information included the general study characteristics (i.e., author name, year of publication, inclusion or exclusion criteria, total number of participants, study design), patient characteristics (baseline NIHSS, time from symptom onset to CTP acquisition), index test (CT scanner, Coverage, Thickness), reference standard and indices of diagnostic performance. We constructed 2×2 contingency tables for true positive cases, false positive cases, false negative cases and true negative cases. If there were no absolute values, we calculated these values from the estimated sensitivities and specificities of each study.

### Statistical analysis and data synthesis

The estimates of sensitivity and specificity and their 95% confidence interval were plotted in paired forest plots. We used a hierarchical summary ROC model (HSROC) which was performed in STATA (version 12) to pool the sensitivity and specificity of CTP in detecting acute ischemic stroke [10].

We included all studies in each pairwise comparison to compare the diagnostic accuracy of CTP with other imaging technologies. We used forest plots to presented the results of the studies which directly compared different index tests. We pooled sensitivities and specificities of the compared different index tests by using bivariate random effects models in SAS 9.0. The model’s parameters were used to plot the ROC curve in RevMan.

We explored heterogeneity by subgroup analyses which used the following pre-specified hypotheses: 1) time between symptom onset and CTP acquisition (≤6 hours vs. 6–24 hours); 2) study design (prospective *vs*. retrospective *vs*. unclear); 3) Specific vascular territory infarction (posterior circulation, anterior circulation infarction).

## Results

**Fig 1:**
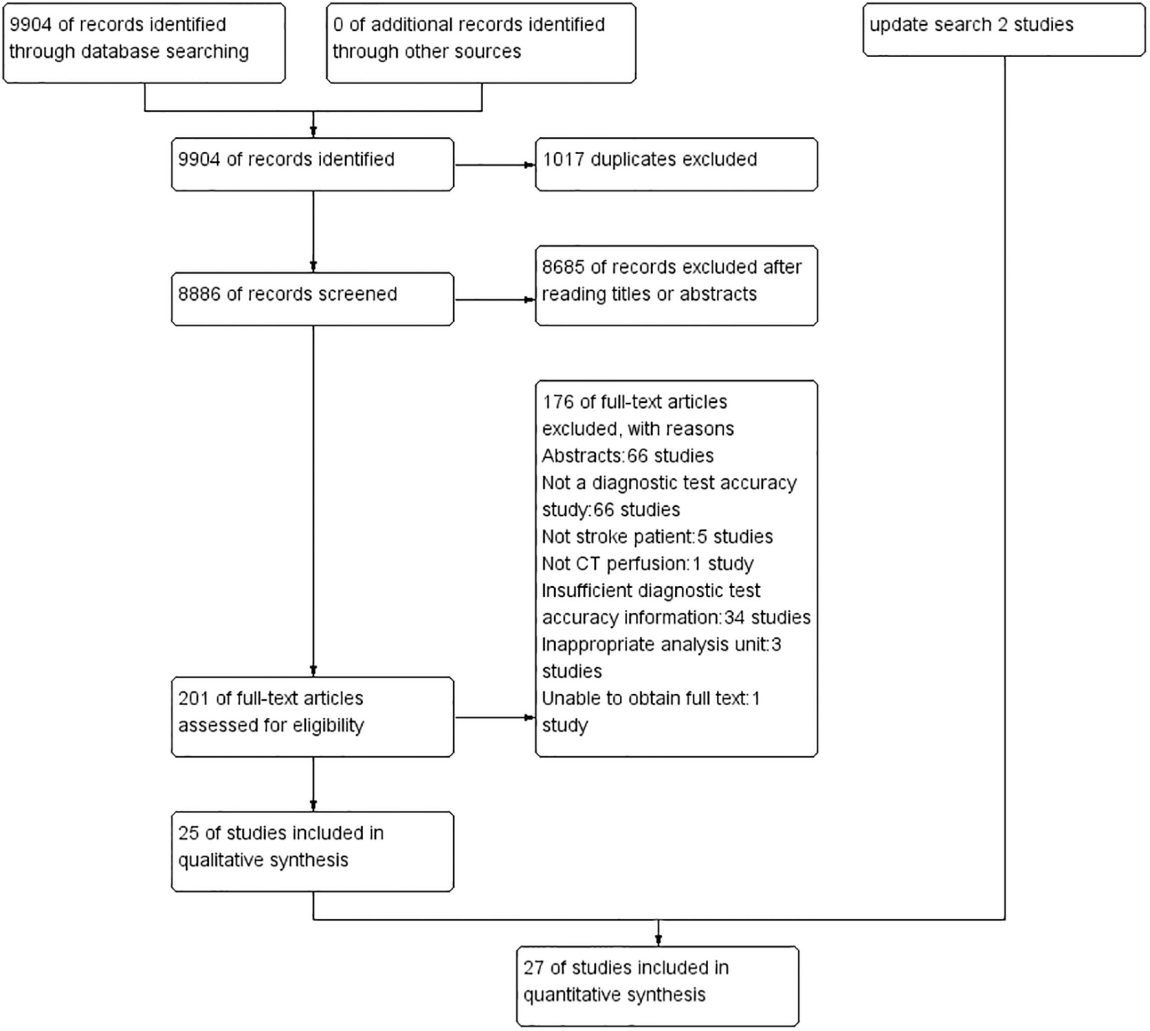
Study flow diagram.

We identified 9904 citations, and 27 citations were included in our analysis after full-text reviews (Fig 1).

We included 27 studies with a total of 2168 sample size. The sample size in the included studies ranged from 10 to 422, and seven of them only included stroke patients (n = 291). All studies were based on a reference standard of follow-up CT or MRI. Thirteen studies stated all patients who underwent CTP within 6 hours after symptom onset. Patients in the other twelve studies underwent CTP in 6–24 hours after symptom onset. Two studies did not report patients’ time of the symptom onset. Twelve studies retrospectively collected patients’ data, eleven studies performed prospective trials and four studies did not describe their study design. The characteristics of included studies were showed in Table 1.

**Table 1:** Characteristics of included studies.

Study	Participants	NIHSS	Stroke	Time hours	Reference Standard	CT scanner	Coverage mm	Thickness mm	Color maps	Design
Campbell 2012[11]	364	10(5–18)	277(76%)	9(3.1)	Follow up CT/MRI	Siemens 16 slice	24	12	TTP, CBV, CBF	Retrospective
Chen 2012[12]	20	NS	20(100%)	20(NS)	Follow up MRI	Toshiba 320 slice	160	0.5	CBV, CBF,MTT, TTP	Prospective
Dai 2009[13]	23	NS	23(100%)	NS	Follow up MRI	Siemens 64 slice	28.8	7.2	CBV, CBF, TTP	Retrospective
Eastwood 2003[14]	15	12.6	14(93%)	8(3.1)	Follow up MRI	GE 1 slice	5–10	5–10	CBV, CBF, MTT	Prospective
Eckert 2010[15]	107	8.3	76(71%)	6(NS)	Follow up CT/MRI	Philips 40 slice	40	10	CBV, MTT	Prospective
Eseban 2004[16]	42	NS	29(69%)	6(NS)	Follow up CT/MRI	GE 16 slice	20	5–10	CBV, CBF, MTT	Retrospective
Hana 2014[17]	87	NS	87(100%)	NS	Follow up MRI	GE 64 slice	NS	NS	CBV, CBF, MTT	Retrospective
Kloska2004[18]	41	10.5	38(93%)	8(3.1)	Follow up CT/MRI	Siemens 4 slice	20	10	TTP, CBV, CBF	Prospective
Koenig 1998[19]	32	NS	28(88%)	6(2.7)	Follow up CT/MRI	Siemens slip-ring	10	10	CBF	Prospective
Langer 2007[20]	50	6(0–28)	38(76%)	8(NS)	Follow up CT	GE multi slice	NS	NS	CBV, CBF, MTT	Prospective
Leng 2003[21]	46	NS	34(74%)	6(NS)	Follow up CT	Maconi multi slice	NS	20	CBF, TTP, MTT	NS
Lin 2009[22]	100	12(4–8)*	65(65%)	3(NS)	Follow up MRI	Siemens16 slice	24	12	TTP, CBV, CBF	Retrospective
Liu 2005[23]	31	NS	31(100%)	24(NS)	Follow up CT/MRI	8 slice	NS	NS	CBV, CBF, MTT	NS
Pepper 2006[24]	15	15(11–20)	14(93%)	6(4.1)	Follow up CT/MRI	Philips 16-slice	24	6	CBV, CBF,MTT	Prospective
Rai2008[25]	422	NS	157(37%)	15(3.9)	Follow up MRI	GE multi slice	20	10	CBV, CBF, MTT	Retrospective
Reichenbach 1999[26]	20	NS	20(100%)	6(2.8)	Follow up CT/MRI	Siemens slip-ring	10	10	TTP	Prospective
Roberts 2001[27]	12	NS	9(75%)	6(NS)	Follow up CT/MRI	GE multi slice	40	10	TTP, CBV, CBF, MTT	NS
Rother2000[28]	22	13.2	20(91%)	6(2.4)	Follow up CT	Siemens slip-ring	10	10	TTP	Prospective
Schramm 2004[29]	22	10(4–8)	13(59%)	6(2.3)	Follow up CT	Siemens multi slice	20	10	TTP, CBV, CBF	Prospective
Sillanpaa 2011[30]	72	7(4–2)	72(100%)	3(NS)	Follow up CT	GE 16 slice, Philips 64 slice	80 or 20	10 or 5	CBV, CBF, MTT	Retrospective
Suzuki 2005[31]	118	NS	110(93%)	10(NS)	Follow up CT/MRI	GE 64 slice	30	10	CBV, CBF, MTT	NS
Wintermark 2005[32]	46	NS	26(57%)	12(5.5)	Follow up CT/MRI	Philips multi slice	40	10	TTP, CBV, CBF, MTT	Retrospective
Youn2008[33]	58	NS	51(88%)	24(3.4)	Follow up MRI	Philips 64 slice	80	10	TTP, CBV, CBF, MTT	Retrospective
Zebedin2011[34]	10	NS	2(20%)	9(NS)	Follow up CT	Toshiba 320-MDCT	5	5	CBV, CBF, MTT	Retrospective
Zhu2011[35]	38	NS	38(100%)	6(NS)	DWI	Philips 64 slice	40	5	CBV, TTP	NS
Sporns2016 [36]	267	4 (4.6–5.3) *	188(70%)	0–9	Follow up MRI	128-slice Siemens dual-source CT	99	5	CBV, CBF,MTT, TTD	Retrospective
van der Hoeven2015[37]	88	2.5 (2–5)*	76(86%)	3.8(2.6)	Follow up CT/MRI	40–320 detector C T(Philips,Siemens, GE, Toshiba)	>40	5	CBF, CBV, MTT	Prospective

* median (IQR)

### Methodological quality of included studies

**Fig 2:**
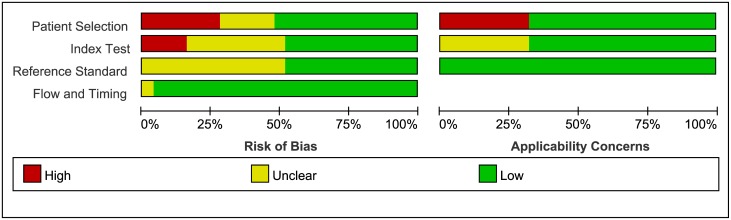
Risk of bias and applicability concerns graph: Review authors’ judgments about each domain presented as percentages across included studies.

**Fig 3:**
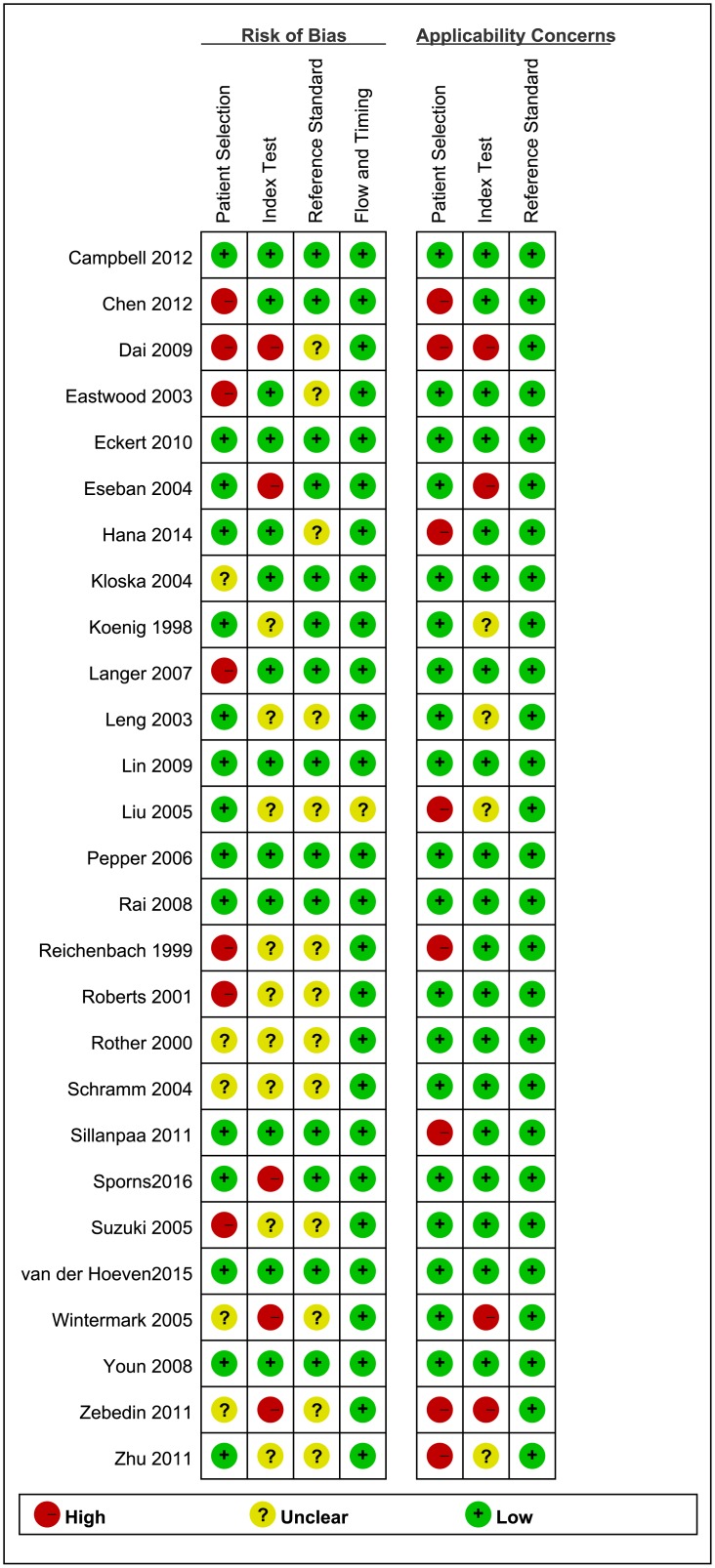
Risk of bias and applicability concerns summary.

The quality of the 27 included studies was varied. Figs 2 and 3 summarized the results of the quality assessment of the included studies. Eight studies were classified as high quality. In the patient selection domain, seven studies were assessed at high risk of bias due to its’ poor reporting of the sampling procedure and the exclusion criteria. Five studies were assessed at unclear risk of bias because those studies were unclearly reported whether the included participants were consecutive or random. Seven studies were of high concern because they only included stroke patients. One study was classified as high concern because the patient cohort comprised only children.

In the index test domain, we considered five studies with high risk of bias, because the retrospective data, and whether the index test results collection and confusion were interpreted without knowledge of the reference standard results. Nine studies were judged with unclear risk of bias for poor reporting of whether the index test results were interpreted without knowledge of the reference standard results. Therefore, these studies were of unclear concern.

Thirteen studies were judged as having unclear risk of bias in reference standard domain, because they failed to report whether the reference standards were interpreted without knowledge of the index test results. However, we assessed these studies as having a low concern in applicability because of their objective results. In twelve studies, not all patients received the same reference standard. The limit time of follow up CT or MRI was unclear. These may have risk of bias in reference standard. However, we assessed these studies as having a low risk of bias in reference standard domain because both CT and MRI were recommended as gold standards by guidelines.

We deemed 26 of the studies at low risk of bias with regard to the flow and timing domain. The remaining study was judged as having an unclear risk of bias because not all patients received the same reference standard.

### Findings

#### Accuracy of CTP in detecting stroke

**Fig 4:**
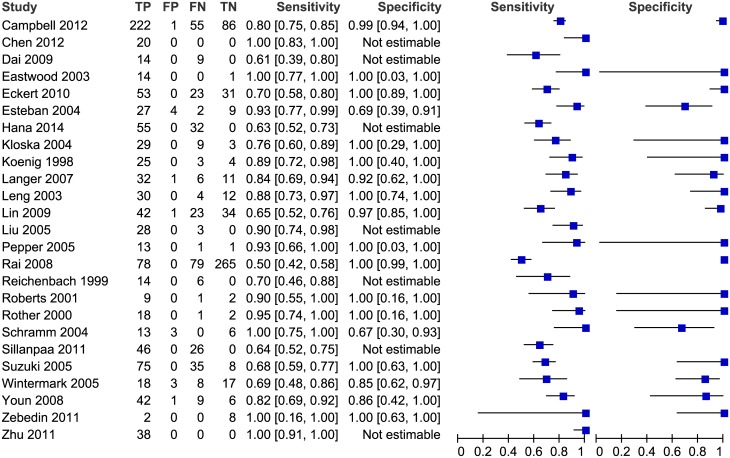
Forest plot of CTP for detection of ischemic stroke. The plot shows study-specific estimates of sensitivity and specificity (with 95% confidence intervals). The studies are ordered according to whether recruitment was prospective or not, and sensitivity. FN: false negative; FP: false positive; TN: true negative; TP: true positive.

**Fig 5:**
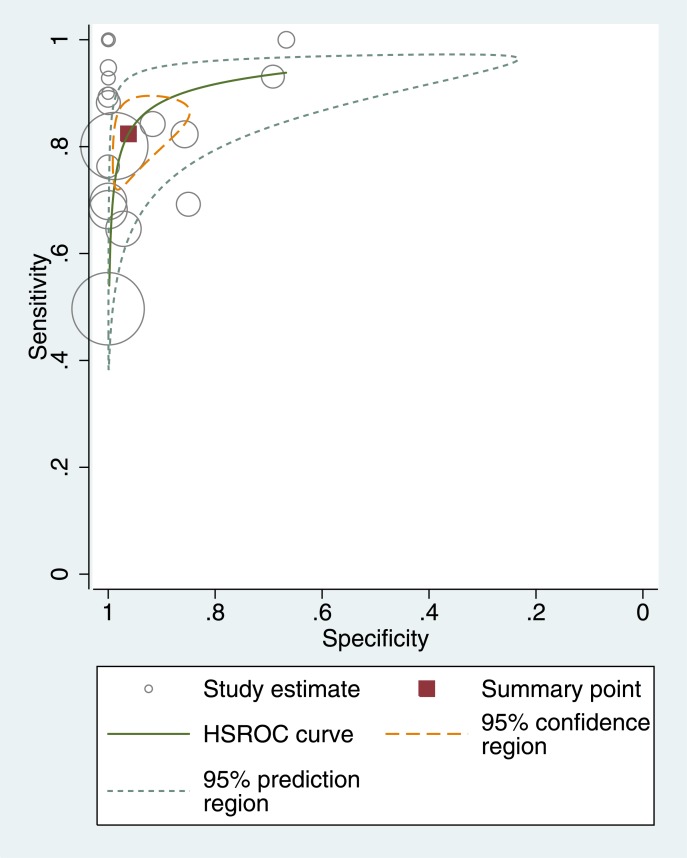
Summary ROC Plot of CTP for detecting ischemic stroke. The circle size represents the sample size of the corresponding study.

Two studies (Sporns2016,van der Hoeven2015) were about posterior circulation, and they were analyzed in subgroup analysis. Fig 4 showed the coupled forest plot of CTP’s sensitivity and specificity in the other twenty-five studies. The sensitivity of CTP in detecting AIS ranged from 50% to 100%, and the specificity ranged from 67% to 100%. Seven studies could not estimate the specificity because no non-disease patients were included, and the pooled sensitivity of these studies was 72% (65–79%). The pooled sensitivity of the remaining 18 studies was 82% (95% CI 75–88%), the specificity was 96% (95% CI 89–99%), the diagnostic odds ratio (DOR) was 118.8 (95% CI 45.6–309.8), the positive likelihood ratio (LR) was 21.8 (95% CI 7.8–60.6) and the negative LR was 0.18 (95% CI 0.13–0.26). Fig 5 shows the accuracy estimates of the 18 included studies in the ROC plot along with summary ROC (SROC) curve.

#### Comparison of CTP and NCCT in detecting stroke

**Fig 6:**
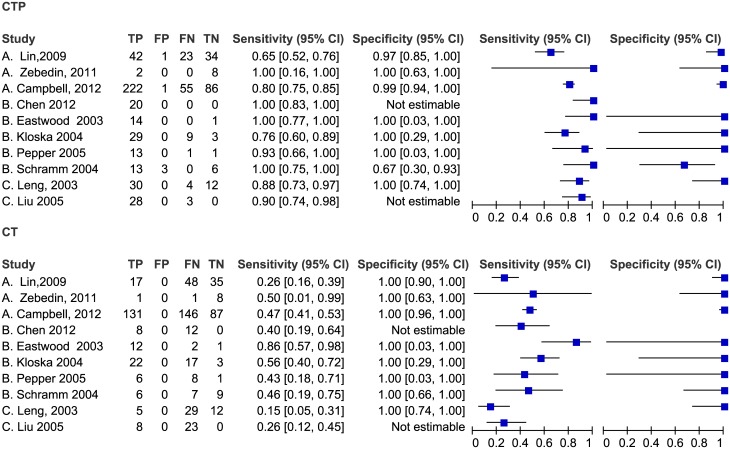
Forest plot of CTP and NCCT for detection of ischemic stroke.

**Fig 7:**
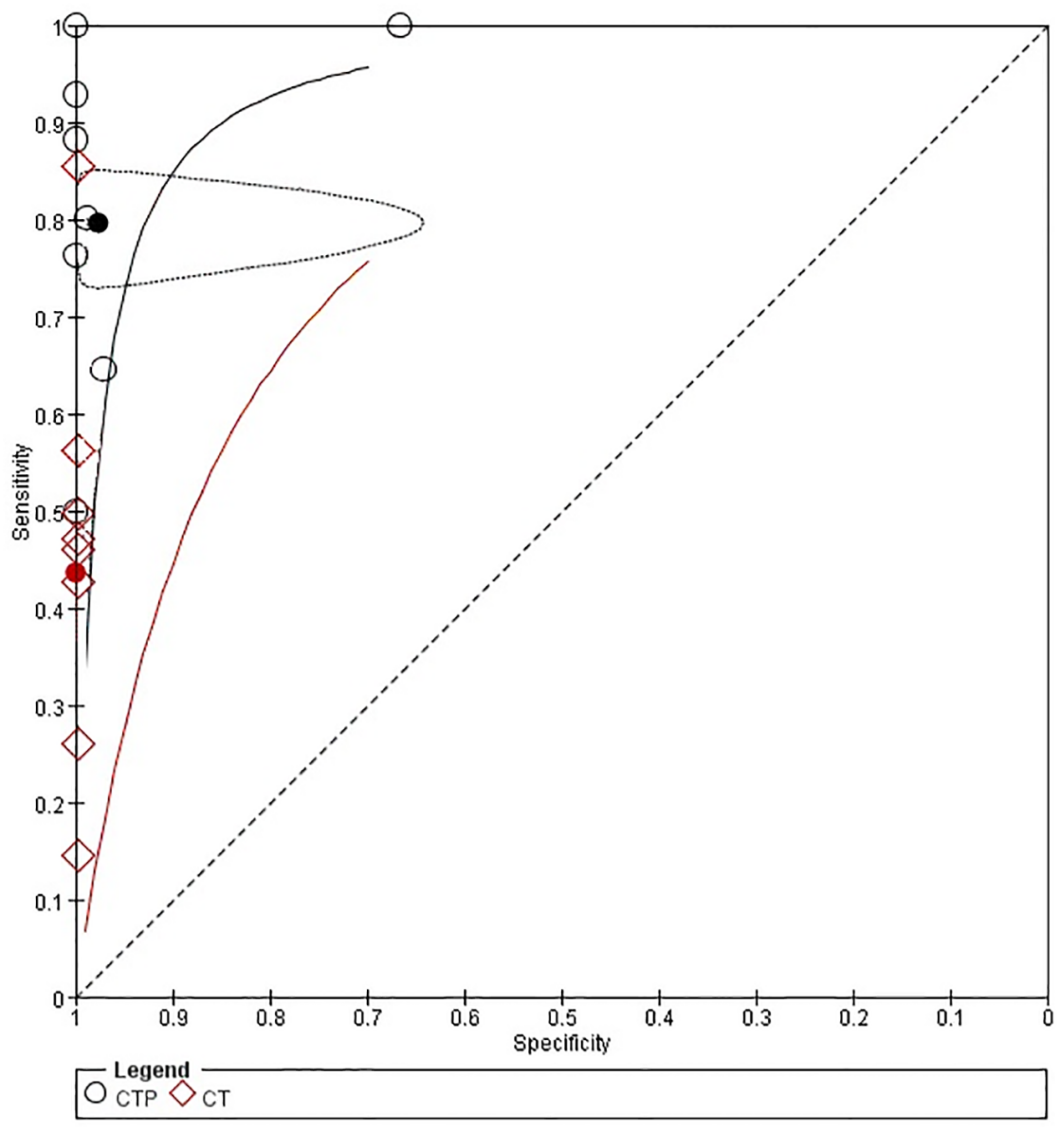
Summary ROC Plot of CTP and NCCT for detecting ischemic stroke. Each ellipse on the plot represents the study estimate of CTP. Each diamond represents the study estimate of NCCT. Red and black solid circles represent the summary sensitivity and specificity for NCCT and CTP respectively, and this summary point is surrounded by a 95% confidence region (dotted line). Red and black solid lines represent HSROC curve of NCCT and CTP respectively.

As shown in Fig 6, ten studies compared the accuracy of CTP with non-contrast CT (NCCT) in detecting acute stroke, and two of these studies were not estimated in specificity. The sensitivity of NCCT ranged from 15% to 86%, while the specificity of NCCT was 100%. All studies showed that the sensitivity of CTP was higher than that of NCCT, and three studies showed a lower specificity of CTP. The pooled sensitivities and specificities of CTP were higher than those of NCCT as shown in Fig 7.

#### Comparison of CTP and CTA in detection stroke

**Fig 8:**
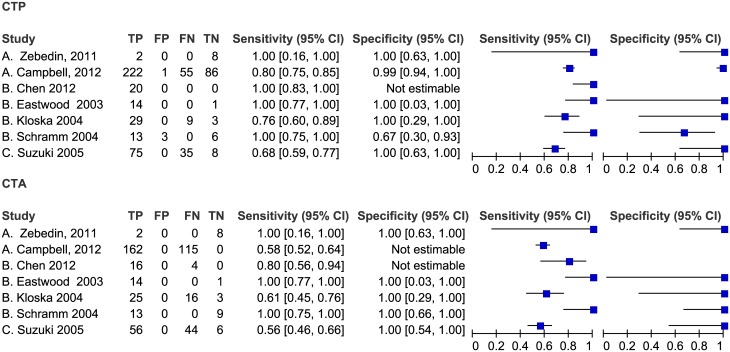
Forest plot of CTP and CTA for detection of ischemic stroke.

**Fig 9:**
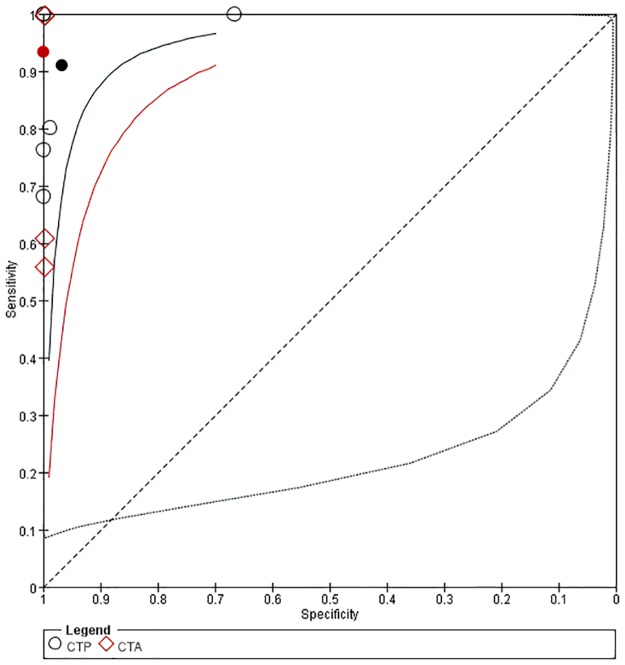
Summary ROC Plot of CTP and CTA for detecting ischemic stroke. Each ellipse on the plot represents the study estimate of CTP. Each diamond represents the study estimate of CTA. Red and black solid circles represent the summary sensitivity and specificity for CTA and CTP respectively, and these summary points are surrounded by a 95% confidence region (dotted line). Red and black solid lines represent HSROC curve of CTA and CTP respectively.

As shown in Fig 8, seven studies compared CTP with CTA directly. The sensitivity of CTA ranged from 56% to100%, and the specificity was 100%. Four studies showed that sensitivity of CTP was higher than that of CTA, and one study showed the specificity of CTP was lower than that of CTA. As shown in Fig 9, the pooled sensitivity of CTP was 91% (95% CI: 56–99%), the pooled specificity was 97% (95% CI: 36–100%), the pooled sensitivity of CTA was 93% (95% CI: 35–99%), and the pooled specificity was 100% (95% CI: 0–100%). There was no significant difference between CTP and CTA in the pooled sensitivities and specificities.

#### Comparison accuracy of CTP in detection acute posterior circulation stroke

Two studies compared the accuracy among CTP, NCCT and CTA in detecting acute posterior circulation stroke. As shown in Table 2, the sensitivity of CTP in detecting acute posterior circulation stroke was higher than that of CTA and NCCT. All of the specificities of CTP, CTA and NCCT were high, and there was no significant difference.

**Table 2:** Accuracy of CTP, NCCT and CTA in detection acute posterior circulation stroke.

Study	Sensitivity(95%CI)	Specificity(95%CI)	TP	FP	TN	FN
CTP						
Sporns2016	77(70–82)	92(84–97)	144	6	73	44
van der Hoeven2015	74(58–86)	93(82–99)	31	3	43	11
pooled	76(70–81)	93(87–97)				
NCCT						
Sporns2016	21(16–29)	96(89–99)	40	3	76	148
van der Hoeven2015	31(18–47)	98(88–100)	13	1	45	29
pooled	23(18–29)	97(92–99)				
CTA						
Sporns2016	44(36–51)	97(91–100)	82	2	77	106
van der Hoeven2015	33(20–50)	98(88–100)	14	1	45	28
pooled	42(35–48)	98(93–100)				

### Subgroup analysis

Results of the subgroup analyses were summarized in Table 3. When we compared prospective study group with retrospective study group, there was no statistically significant difference in sensitivity (p>0.05), but there was significant difference in specificity (p<0.05). Differences in the sensitivity and specificity of CTP were also not statistically significant between the ≤6 hours study group and 6–24 hours study group (p>0.05).

**Table 3:** Subgroup analyses in different study design and time from symptom onset to CTP acquisition.

Subgroups	Studies	Sensitivity, % (95%CI)	Specificity, % (95%CI)
Study design			
Prospective	10	81(76–86)	94(85–98)
Retrospective	10	69(66–72)	98(96–99)
Unclear	5	81(75–86)	100(85–100)
Time from symptom onset to CTP acquisition			
≤6 hours	12	78(74–82)	93 (86–97)
6–24 hours	11	73(70–76)	99(97–99)

## Discussion

### Accuracy of CTP in diagnosing acute ischemic stroke

CTP had high sensitivity and very high specificity in detecting ischemic stroke. The pooled sensitivity and specificity of CTP in this systematic review were both higher than those of previous systematic review which included 15 studies [7]. In our study, CTP had higher sensitivity than NCCT and had similar accuracy to CTA in diagnosing AIS. However, CTA provides high diagnostic accuracy in detecting site of occlusion while CTP offers high diagnostic accuracy in distinguishing between infarcted and ischemic penumbra [8]. The study by Tan also found that CTA source images performed better than CTP in detecting the site of occlusion (sensitivity,94.6%; specificity, 100.0% vs sensitivity, 88.2%; specificity, 95.3%), while CTP performed better than CTA source images in prediction of the anatomic distribution of the final infarct(sensitivity, 80.4%; specificity, 96.8% vs sensitivity,72.0%; specificity, 98.4%) [38]. In the 195 specified false negative cases, 106 (54%) were cases of lacunar infarct, 56 (29%) were cases that not covered by CTP, eleven (6%) were cases of atherothrombotic infarction, nine (5%) were cases of territorial infarct, five (3%) case were motion artifacts, four (2%) case were due to technical failure, two (1%) were cases of reperfusion, and two (1%) were cases of cardioembolism. Therefore, some of the patient characteristics (especially the percentage of lacunar infarct patients and the coverage of CTP imaging) impacted the sensitivities of CTP. There were some ways to improve the accuracy of CTP, such as the toggling-table technique could double brain coverage, and the latest generations of CT scanners could provide whole brain coverage [12, 27, 33].

### Factors which affect the accuracy of CTP in diagnosing acute ischemic stroke

The influence of study design in the diagnostic accuracy of CTP was small. The pooled CTP sensitivity of 9 prospective studies was higher than that of 8 retrospectively studies, but there was no significant difference. The accuracy of CTP was not correlated with the length of time after symptom onset. The sensitivity of group which underwent CTP scan within 6 hours from symptom onset was higher than that of the 6–24 hour group, but the difference was not statistically significant. The result was similar to the study by Hana [17].

There were no defined CTP processing parameters, biologically salient perfusion parameters and thresholds for decision making, thus different CTP protocols, image processing & analysis methods, and color maps (CBV, CBF, MTT, and TTP) maybe used in various studies, and all of these could vary the sensitivity of CTP [39–43].

The severity of the stroke and size of infarct also influenced both the sensitivity and specificity of CTP. The study by Hana found that CTP had higher sensitivity when the size of infarction was over 3 cm^2^ (90% vs 29%, P<0.001)[17]. Another study by Huisa found that sensitivity and specificity of CTP were increased to 81% and 91% when they selected MCA territory strokes >5cc [44]. The study by Eckert showed that the sensitivity of CTP was higher in group which with average NIHSS = 8.3 than in group which with NIHSS = 4.4 (100% vs 42.6%)[15]. The study by Rother showed that the sensitivity of CTP in group with NIHSS>10 was higher than sensitivity of CTP in group with NIHSS<10(100% vs 33%)[28]. Another study by Campbell also found that the sensitivity of CTP was lower in patients with NIHSS≤7 than that in patients with NIHSS = 10 (63% vs 80%)[11]. In twelve included studies which reported average NIHSS, only two studies had average NIHSS≤4, thus the results of this review may overestimated.

In our study, the pooled sensitivity and specificity of CTP in diagnosing AIS were similar to those in systematic review by Biesbroek. Compared with Biesbroek’s study, our systematic review included other ten studies. The sensitivity and specificity of CTP in the systematic review by Sabarudin were lowest, because the included criteria of Sabarudin’s study were unclear and some studies were missed, but these missed studies were included in our review.

### The safety of CTP in diagnosing acute ischemic stroke

Renal damage and radiation exposure were two disadvantages of CTP. One included study reported that two patients had developed into subcutaneous contrast extravasation, but no clinically significant renal dysfunction was reported [11]. In the available literature, patients with normal renal function who had CTP had low risk of renal dysfunction. CTP had more additional radiation dose than NCCT. However, the newest scanners with optimized protocols can image the entire cranium without substantial increases in radiation dose. All included studies did not report the safety of CTP except two studies that reported the administered radiation doses [33,34] which were lower than the recommended dose of 2.0–3.4 mSv described in the Hoeffner study [45]. The effective dose of CTP was 1.1/1.2 to 5 mSv, and it was not higher than NCCT, but it was less than CTA [46–49].

### Strengths and weaknesses of the review

This research not only review the accuracy of CTP in detecting ischemic stroke, but also review the comparative accuracy of CTP in detecting ischemic stroke, and the latter was the most difference compared to previous reviews. We included directly comparative studies that evaluated two index tests in the same group of patients, and these studies provided the best evidence in comparing the accuracy of imaging technologies [50]. In these included studies, the characteristics of the patient varied, and patients in half of the studies may not be representative. Some of the included studies had very small sample size, and it might influence the estimation accuracy. Thirteen studies did not report whether the investigators were blinded to the results of reference standard test and relevant clinical information, and it might overestimate the accuracy of CTP. Only eight of twenty-seven studies had low risk of bias. Like other systematic reviews of the diagnostic test accuracy, the heterogeneities of the sensitivity and specificity in the included studies were high that may impact the reliability of the pooled results.

## Conclusion

CTP is more accurate than NCCT and has similar accuracy to CTA in detecting acute ischemic stroke. However, the evidence is not strong. There is potential benefit of using CTP to select stroke patients for treatment, but more high-quality evidence is needed to confirm this result.

## Supporting information

S1 Table Search strategy. (DOCX)

S2 Table PRISMA checklist applied to this review. (DOC)
