# Supplementary material for: Comparative accuracy of CT perfusion in diagnosing acute ischemic stroke: A systematic review of 27 trials
Source: PLoS One. 2017 May 17;12(5):e0176622. doi: 10.1371/journal.pone.0176622 (PMC5435168; doi:10.1371/journal.pone.0176622)
Supplement: S1 Table — (DOCX) [file pone.0176622.s001.docx]

EMbase

#1 'spiral computer assisted tomography'/exp

#2 'multidetector computed tomography'/exp

#3 'scintigraphy'/exp

#4 perfusion:ab,ti

#5 'computer assisted tomography':ab,ti

#6 'computed tomography':ab,ti

#7 'brain angiography'/exp

#8 'brain perfusion'/exp

#9 #1 OR #2 OR #5 OR #6

#10 #3 OR #4 OR #7 OR #8

#11 #9 AND #10

#12 'perfusion ct':ab,ti

#13 'ct perfusion':ab,ti

#14 #11 OR #12 OR #13

#15 'cerebrovascular disease'/exp

#20 stroke*:ab,ti

#21 apoplex*:ab,ti

#22 'cerebral vascular':ab,ti

#23 cerebrovasc*:ab,ti

#24 #20 OR #21 OR #22 OR #23

#25 brain:ab,ti

#26 cerebell*:ab,ti

#27 vertebasilar*:ab,ti

#28 hemisphere*:ab,ti

#29 intracran*:ab,ti

#30 intracerebral*:ab,ti

#31 infratentorial:ab,ti

#32 'anterior circulation':ab,ti

#33 ischaemi*:ab,ti

#34 infarct*:ab,ti

#35 emboli*:ab,ti

#36 thrombo*:ab,ti

#37 occlus*:ab,ti

#38 hypoxi*:ab,ti

#39 #33 OR #34 OR #35 OR #36 OR #37 OR #38

#40 #25 OR #26 OR #27 OR #28 OR #29 OR #30 OR #31 OR #32

#41 #39 AND #40

#42 #15 OR #24 OR #41

#43 #14 AND #42
